# Knowledge, attitude and practice of community drug distributors’ about onchocerciasis and community directed treatment with ivermectin in Quara district, North Western Ethiopia

**DOI:** 10.1186/s13104-016-2010-x

**Published:** 2016-04-06

**Authors:** Fitsum Weldegebreal, Girmay Medhin, Zemichael Weldegebriel, Mengistu Legesse

**Affiliations:** Department of Medical Laboratory Science, College of Health and Medical Science, Haramaya University, Harar, Ethiopia; Aklilu Lemma Institute of Pathobiology, Addis Ababa University, Addis Ababa, Ethiopia; Metema Hospital, Gendwuha, North Gondar, Amhara Region Ethiopia

**Keywords:** Onchocerciasis, Knowledge, Attitude, Practice, Quara, Ethiopia

## Abstract

**Background:**

Onchocerciasis is one of the most important public health problems over large areas of tropical Africa countries including Ethiopia. The African Program for Onchocerciasis Control (APOC) has been working with ultimate goal of reducing the public health and socio-economic problems of onchocerciasis through administration of the tablet for continuous 12–15 years using the strategy of yearly community-directed treatment with ivermectin (CDTI) in endemic areas of Africa to kill the microfilariae that invade the eyes and are present in the skin to be transported to another victim by the black fly. The objective of this study was to assess knowledge, attitude and practice of community drug distributors (CDDs) towards onchocerciasis and CDTI in Quara district.

**Result:**

Of all the study participating CDD 11.4 % (9/79) said that they knew about the etiology of the disease, 35.4 % (28/79) had good level of knowledge, 19 (24.1 %) had good level of positive attitude and 18 (22.8 %) had good level of positive practice about onchocerciasis. Similarly, 45.6 % (36/79), 81.0 % (64/79) and 29.1 % (23/79) had good level of knowledge, attitude and practice about CDTIP, respectively. Being a female CDD (adjusted OR 7.246, P = 0.035, 95 % CI 1.147, 45.455) and being older than 35 years (adjusted OR 8.435, P = 0.001, 95 % CI 4.53, 9.003) were significantly associated with the likelihood of having good level of knowledge about the disease.

**Conclusion:**

Although onchocerciasis is endemic in Quara district, large proportion of the CDDs had misconceptions about its causation, transmission and prevention. Therefore, CDTIP for onchocerciasis control need to be supported by proper and continuous training, and health education about different aspects of the disease.

**Electronic supplementary material:**

The online version of this article (doi:10.1186/s13104-016-2010-x) contains supplementary material, which is available to authorized users.

## Background

Onchocerciasis, commonly known as ‘river blindness’, is a debilitating vector-borne disease caused by a parasite, *Onchocerca volvulus* [1, 2] and transmitted by the bite of black-fly, *Simulium damnosum*, which breeds in fast flowing streams and rivers [3]. Adult worms dwell in subcutaneous nodule, from where fertilized females produce millions of microfilariae (mf) which live and freely migrate in the intercellular spaces of the skin tissues [4]. The disease is characterized by causing skin lesions with severe itching, a serious eye lesion and blindness known as river blindness. It is a chronic and slowly progressive disease. The initial infestation often occurs in childhood, and many of the affected individuals remain asymptomatic for long periods. The disease is a major problem among rural communities living in close proximity to rivers in Sub-Saharan African countries [5].

Onchocerciasis is one of the most important public health problems and widespread parasitic disease over 30 countries of tropical Africa and in six countries of Latin and Central America and one country in the Arabian Peninsula. About 125 million people in the world are estimated at risk of the infection and the majorities of them (96 %) are found in Africa [5, 6]. In Ethiopia, 3 million people are already infected, whereas 7.3 million are at risk of infection and almost everyone in an endemic village will harbor the disease. Nine regions surveyed for river blindness were shown to be endemic; the endemic areas extend from the northwest part to southwest part of the country that borders Sudan [7, 8]. Mass treatment of high risk communities with ivermectin was adopted in line with the African Program for Onchocerciasis Control (APOC) community directed treatment with ivermectin (CDTI) strategy [9, 10]. The Right to Sight global initiative was launched in 1999 by the World Health Organization (WHO) and The International Agency for the Prevention of Blindness (IAPB), with the aim of eliminating avoidable blindness by the year 2020 and in so doing preventing an estimated 100 million people from going blind. An indicator of the impact of VISION 2020 is whether the prevalence of avoidable blindness is indeed declining [11].

The main control strategy for onchocerciasis in Ethiopia is mass treatment with ivermectin [12]. The ultimate goal of the APOC is to reduce the public health and socio-economic problems of onchocerciasis by providing the administration of the tablet for a period of 12–15 years using the strategy of yearly CDTI in endemic areas to kill the microfilariae that invade the eyes and are present in the skin to be transported to another victim by the black fly. Ivermectin is highly effective in the mass treatment of onchocerciasis, it kill the microfilariae that invade the eyes and are present in the skin to be transported to another victim by the black fly. Which results, keeping the microfilariae population down, transmission and disease effects are minimized for such a period of time that the adult worms eventually die off with in the period of 12–15 years CDTI program [13].

The successful use of ivermectin at a national scale requires a broad public health program designed to ensure appropriate distribution, monitoring, community education, and record keeping. In Latin America, and specifically Guatemala, additional opportunities to control onchocerciasis exist and it indicates that interruption of transmission, eventually resulting in local elimination of the disease, could be feasible [14, 15].

Based on the REMO results of 1997 a community-based free distribution of Ivermectin was first launched in Sheka Zone of Southwestern Ethiopia in the year 2001 and scaled up to the other endemic regions of Ethiopia phase-by-phase [16]. CDTI program was introduced to Quara district in 2003 by WHO/APOC in partnership with Federal Ministry of Health (FMOH), the Carter Centre, the local administration and the communities with 100 % geographical coverage and all eligible peoples addressed by CDD’s [16]. All eligible members of the community in the District have been treated with ivermectin in campaign once a year by CDDs [17]. Except pregnant women, less than 1 week of lactating mothers, seriously ill individuals and under five children, any individual living in the selected area is eligible for the CDTIP. This being the actual practice the knowledge of community’s about onchocerciasis and community’s attitude towards the CDTIP has not been studied. To attain community participation and design socially/locally acceptable control strategies, health program planners and implementers must be familiar with CDD’s knowledge and attitude in relation to onchocerciasis [18–20]. The successful use of ivermectin at national, regional, zonal, district and kebeles level requires a broad public health program designed to ensure appropriate distribution, monitoring, community education, and record keeping. Since there is paucity of information as few studies have been carried out to understand these issues, a study aimed at assessing factors affecting the sustainability of the program has great importance to generate information on the awareness, challenges and obstacles faced during ivermectin distribution, and design appropriate strategy to improve the outcomes. *One of the important reason push us to conduct this research was the overall knowledge and beliefs of the community about the disease of onchocerciasis and the CDTIP were in our research conducted were low* [21]. *To intervene this knowledge and attitude gap of the community knowing the knowledge of the CDDs about onchocerciasis and their attitude towards the CDTIP is crucial*. The finding of this study will serve as an input for the Quara District Health Office while designing monitoring and evaluation of the on-going onchocericiasis control program. The information obtained from the study provides a basis for understanding how best to sustain community control and to achieve success in the control of onchocerciasis as a public health and socioeconomic problem in the study area. However, the knowledge of the CDDs about onchocerciasis and their attitude towards the CDTIP has not been studied in the present study area. Therefore, the aim of this study was to investigate CDD’s knowledge and beliefs about onchocerciasis and their attitudes towards the CDTIP in Quara area, North Western Ethiopia.

## Methods

### Description of the study area and population

Between October 2012 and January 2013, a community-based cross sectional survey was conducted in Quara district, north western Ethiopia, which is located 1041 km North of Addis Ababa and 324 km North-West of Gondar town. The district has a total area of 858,580 km^2^ and it shares geographical boarder with Metema District in the North, Benshangul Regional State in the South, North Sudan in the West, Alefa district in East and Awi zone in South West. The three major ethnic groups in the district are Aguw, Amhara and Gumuz. There are also minorities’ ethnic groups who come from Tigray, Oromiya and the Southern Nations Nationalities and Peoples Region (SNNPR). Based on the 2007 national housing and population census [22], the district has 19 kebeles (small government administrative units) with a total population of 93,629 of which 49,750 are males. The district has 20, 806 households with an estimated density of 4.50 person per square kilometer [20]. Seventeen kebeles are occupied by the Amhara ethnic group and two kebeles are occupied by the Aguw and Gumuz ethnic groups. There are five health centers and 28 health posts that provide routine health services to the population of the district. Onchocerciasis is one of the major public health problems in the District [17] with the prevalence of 6.9 % [23].

### Sample size estimation and data collection

Of the 17 kebeles, two kebeles are occupied by Amhara ethnic group were randomly selected, while the two kebele occupied by the Aguw and Gumuz ethnic groups were both included in this study. All CDDs’ (n = 87) residing in the selected four kebeles and consented to participate in the study were included in the study. The CDDs’ were eligible if they were resident of the selected four kebeles, at least 18 years of age, and willing to consent to participate in the study. CDD’s registration book of each kebele was used to recruit the study participants. Structured questionnaires Additional file 1 were prepared in English based on information from available literatures [18, 19, 21, 24, 25] and the questionnaire were translated into Amharic and pre-tested for clarity and cultural acceptability in the district. The participants were interviewed in their local languages by trained data collectors (health extension workers) who speak the local languages. Each interview was conducted through house to house visit. Information on the socio-demo-graphic characteristics of the participants was also included in the questionnaire.

### Ethical consideration

The study protocol was approved by the Ethical Clearance Committee of Aklilu Lemma Institute of Pathobiology (ALIPB), Addis Ababa University. Permission was obtained from Quara District Health Office and from the four selected kebeles administrators. Participants were informed about the objective of the study and they were assured the confidentiality of the data to be maintained. Informed written consent was obtained from all participants prior to data collection.

### Data analysis

The collected data were double entered into a data entry file using EpiData software, V.3.1. The data were transferred to SPSS soft-ware V.16 for anaysis. Pearson Chi square was used to evaluate the statistical significant of bivariate association of selected covariate. Odds ratio with 95 % CI generated using logistic regression were used to describe the strength of association between the selected study variables (i.e. outcome and independent variables) before and after controlling for possible confoundering variables. Bivariate and multivariable logistic regression analysis were performed to explore independent variables that were predictors of overall knowledge (causative agents, sign/symptoms, mode of transmission, treatment and preventive methods of onchocerciasis), attitude and practice of the CDDs on onchocerciasis and CDTIP. For each questions the correct answer provided by the respondent was coded as one and wrong answer provided by the respondent was coded as 0. Score of all correct responses were added to generate the overall knowledge, attitude and practice scores. Respondents whose knowledge, attitude and practice scores equal and greater than the mean were considered as having ‘good knowledge, positive attitude and good practice’ while those below the mean were considered as having ‘poor knowledge, attitude and practice’. The criterion for significance was set 5 %.

## Results

### Socio-demographic characteristics of the study participants

Out of 86 CDDs residing in the study kebeles, 79 CDDs participated in the study resulting in response rate of 91.8 % and male respondents constituted of 84.8 % (57/79). The age of the CDD respondents ranged from 18 to 88 years with mean age of 34.7 (SD = 10.4) years. The majorities were in the age range of 25–49 years, and were belonged to Amhara ethnic group, farmers and followers of Orthodox Christianity (Table 1).

**Table 1:** Socio-demographic characteristics of CDD respondents (n = 79), Quara district, 2013

Characteristics	Response	Number (%)	Characteristics	Response	Number (%)
Kebeles	Yikaho	13 (16.5)	Marital status	Unmarried	67 (84.8)
	Mahdid	11 (13.9)		Married	11 (13.9)
	Bambaho	26 (32.9)		Others	1 (1.3)
	Dubaba	29 (36.7)	Religion	Orthodox	70 (88.6)
Gender	Male	67 (84.8)		Muslim	9 (11.4)
	Female	12 (15.2)	Educational level	Primary (1–8)	71 (89.9)
Age in years	18–24	10 (12.7)		Secondary	8 (10.2)
	25–49	65 (82.3)	Occupation	Farmer	69 (87.3)
	50+	4 (5.1)		Others	10 (12.7)
Ethnic group	Amhara	55 (69.6)	Family size	1–4	43 (54.4)
	Agewu	13 (16.5)		5+	36 (45.6)
	Gumuz	11 (13.9)			

### Knowledge, attitude and practice of the CDDs about onchocerciasis

The level of knowledge, attitude and practice of respondents is summarized in Tables 2, 3 and 4. All respondents have heard about onchocerciasis (‘wara’) and 11.4 % (9/79) said that they knew about the etiology (causative agent) of the disease, which was named as filarial worm. The majority of the respondents’ 88.6 % (70/79) had at least one misconception about the causative agent of onchocerciasis including poor personal hygiene, living in poor environmental sanitation and sun scorching. Sixty three (79.9 %) of the study respondents mentioned onchocerciasis can be transmitted, of whom 72.2 % (57/79) knew that the transmission is related to black fly biting. The rest of the respondents (27.8 %) had misconceptions about mode of transmission of onchocerciasis including Black (river) fly, contact with a person who has the disease, mosquito bite, sharing cloths, sexual contact and through breath. Seventy four respondents (93.7 %) mentioned that onchocerciasis is treatable. Regarding its symptoms, 78.5 % mentioned itching, 40.5 % mentioned skin change and 39.2 % mentioned edema (Table 2).

**Table 2:** Knowledge of CDDs (n=79) about onchocerciasis, Quara district, 2013

Indicative questions on knowledge	Response	Number (%)
Have you ever heard about the disease called onchocerciasis	Yes	79 (100.0)
	No	0 (0.0)
The cause of the disease	Filarial worm	9 (11.4)
	Black (river) fly	57 (72.2)
	Mosquito	2 (2.5)
	Living in poor environmental sanitation	3 (3.8)
	Poor personal hygiene	4 (5.1)
	Sun scorching	2 (2.5)
	Being not vaccinated	2 (2.5)
Oncho transmits from person to person	Yes	63 (79.9)
	No	11 (13.9)
	I do not known	5 (6.3)
The mode of transmission of the disease	Black fly bite	57 (72.2)
	Contact with infected persons	1 (1.3)
	Mosquito bite	1 (1.3)
	Through breath	2 (2.5)
	Sharing clothes	1 (1.3)
	Sexual contact	1 (1.3)
The signs and symptoms of the disease	Itching	62 (78.5)
	Edema	31 (39.2)
	Skin change	32 (40.5)
Oncho is preventable disease	Yes	78 (98.7)
	No	0 (0.0)
	I do not know	1 (1.3)

**Table 3:** Attitude and practice of CDD respondents (n=79) about onchocerciasis, Quara district, 2013

Indicative questions on attitude and practice	Response	Number (%)
Have you/your families ever been sick from Onchocerciasis	Yes	16 (20.3)
	No	62 (78.5)
	I do not remember	1 (1.3)
Is onchocerciasis a serious disease	Yes	75 (94.9)
	No	4 (5.1)
Do you think onchocerciasis needs treatment	Yes	74 (93.7)
	No	4 (5.1)
	I do not know	1 (1.3)
What type of treatment	Modern	74 (100.0)
	Traditional	0 (0.0)
If modern, which drug is needs to treat the disease	Ivermectin/mectizan	74 (100.0)
	Albendazole	0 (0.0)
Do you think onchocerciasis is preventable disease	Yes	78 (98.7)
	No	0 (0.0)
	I do not know	1 (1.3)
What do you do to prevent Onchocerciasis	Avoiding river bathing	
	Yes	31 (39.7)
	No	47 (60.3)
	Wearing protective clothes	
	Yes	38 (48.7)
	No	40 (51.3)
	Taking drug	
	Yes	43 (55.1)
	No	35 (44.9)
	Using bed net	
	Yes	30 (38.5)
	No	48 (61.5)
	Environmental sanitation	
	Yes	22 (28.2)
	No	56 (71.8)
	Personal hygiene	
	Yes	7 (9.0)
	No	71 (91.0)
If your answer for the above question is wearing protective clothes, in what way is used	In the lower extremities (below the knees)	34 (89.5)
	Around head and shoulders	4 (10.5)

**Table 4:** Good level of knowledge, attitude and practice of CDD respondents (n = 79) about onchocerciasis, Quara district, 2013

Outcome variables	Performance on the outcome scores		
	Mean score	Number (%) below mean score	Number (%) equal and above mean score
Knowledge of CDDs	3.52	51 (64.6)	28 (35.4)
Attitude of CDDs	0.37	60 (75.9)	19 (24.1)
Practice of CDDs	2.16	61 (77.2)	18 (22.8)

Among the total respondents, 94.9 % believed that onchocerciasis is a serious disease, 98.7 % said that onchocerciasis is preventable and the preventive methods are drugs (55.1 %), wearing protective cloths (48.7 %), avoiding river bathing (39.7 %) and use of bed net (38.5 %). All the participants mentioned that onchocerciasis is treatable and also knew the name of modern medicine or drug used to treat the disease (i.e. ivermectin/mectizan). About 20.3 % of the CDD respondents reported that their families had got the disease (Table 3).

Generally, in this study, majority of the study participants had poor level knowledge of onchocerciasis (i.e. only 35.4 % of the participants had good level of knowledge). Similarly, majority of the study subjects had poor attitude and practice about onchocerciasis (i.e. only 24.1 and 22.8 %) study subjects had good attitude and good practice on onchocerciasis, respectively (Table 4).

### CDD’s knowledge, attitude and practice about CDTI

Among the respondents, 65 (82.3 %) mentioned that they distributed the drug by moving house to house, while 38 (48.1 %) mentioned that the drug has serious side effects and 19 (24.1 %) knew individuals who interrupted the treatment due to the reasons including fear of the drug side effect and not present during the drug distribution period (Table 5).

**Table 5:** Knowledge, attitude and practice (KAP) of CDD respondents (n = 79) towards CDTI, Quara district, 2013

Indicative questions on knowledge, attitude and practice	Response	Number (%)
Distribution mechanism	Center place	14 (17.7)
	House to house	65 (82.3)
Are you voluntary to serve the community	Yes	77 (97.5)
	No	2 (2.5)
If no for the above ques reason for not serving	Lack of support from government	1 (50.0)
	Lack of commitment	1 (50.0)
How is the coverage of drug distribution in the village	100 %	66 (83.5)
	Partially	13 (16.5)
Does the drug have serious side effects on the community	Yes	38 (48.1)
	No	28 (35.4)
	I don’t know	13 (16.5)
If your answer for the above ques yes, how do you detect	From Individual report	21 (55.3)
	Making round visit	17 (44.7)
Is there anyone who interrupted the treatment	Yes	19 (24.1)
	No	49 (62.0)
	I do not know	11 (13.9)
If your answer for the above que. is yes, what was the reason for interrupting the treatment?	Fear of the side effect of the drug	14 (73.7)
	Not present during the distribution period	5 (26.3)
The status of community participation	High	77 (97.5)
	Low	2 (2.5)
The perception of the community for CDTI	Very good	77 (97.5)
	Poor	2 (2.5)
What is your recommendation for continuity of the program	Incentive is needed	65 (69.9)
	Support from health profession	28 (29.1)

Of the total respondents, 77 (97.5 %) of them served in the community voluntarily. For sustainability of this program, 65 (69.9 %) of the study participants recommended having incentive, but 28 (29.1 %) recommended support from health profession (Table 5). Among the respondents, 66 (83.5 %) indicated that the coverage of drug distribution in the village is 100 %. Twenty one (55.3 %) of the study participants detected the drug side effect from individual self-report and 17 (44.7 %) detected during house to house round visit. Seventy seven (97.5 %) respondents indicated that the status of the community participation and the perception of the community in the program are high (Table 5). From the total respondents; 36 (45.6 %), 64 (81.0 %) and 23 (29.1 %) had good level of knowledge, attitude and practice about CDTI, respectively (Table 6).

**Table 6:** Good level of knowledge, attitude and practice of CDD respondents (n = 79)about CDTI, Quara district, 2013

Outcome variables	Performance on the outcome scores		
	Mean score	Number (%) below mean score	Number (%) equal and above mean score
Knowledge of CDDs	2.41	43 (54.4)	36 (45.6)
Attitude of CDDs	0.85	15 (19.0)	64 (81.0)
Practice of CDDs	0.41	56 (70.9)	23 (29.1)

### Results from logistic regression analysis

In bivariate analysis, male CDDs respondents were approximately 7 times more likely to have good level of knowledge compared to female CDDs (adjusted OR 7.247, P = 0.035, 95 % CI 1.147, 45.455), and CDDs who are at least 35 years of age are about 8 times more likely to have good level of knowledge compared to CDDs who are younger than 35 years of age (adjusted OR 8.435, P = 0.001, 95 % CI = 4.53, 9.003). CDDs individuals who served 5 years and above were approximately four times more likely to have good knowledge than individuals who served less than 5 years **(**adjusted OR 3.710, P = 0.001, 95 % CI = 2.101, 11.201). The statistical significance of age, serves years CDD and sex with good level of knowledge was also maintained in the fully adjusted model (Table 7).

**Table 7:** Results from logistic regression analysis about onchocerciasis

Variable knowledge	Crude odds ratio, 95 % CI	P value
Gender		
Female	1	0.035
Male	7.247 (1.147, 45.455)	
Age		
Below 35 years	1	0.001
35 years and above	8.435 (4.53, 9.003)	
Services year of CDDs		
Served less than five years	1	0.001
Served five years and above	3.710 (2.101, 11.201)	

## Discussion

All of the CDDs who participated in the present study are familiar with onchocerciasis which could probably be due to the endemicity of the disease in the study area and because they are the key in the distribution of the drug for more than 5 years. However, only 11.4 % knew about the etiology (causative agent) of the disease. The majority of the respondents associated the cause of the disease with the bite of black flies, poor personal hygiene, living in poor environmental sanitation, and Sun scorching. This is consistent with the study conducted in Homa District, Western Ethiopia [20].

A few of CDD held at least one misconception about mode of transmission of onchocerciasis including contact with a person who has the disease, mosquito bite,sharing cloths, sexual contact and through breath. This is consistent with the study conducted in Homa District, Western Ethiopia [20].

Generally in this study, majority of the study participants had poor knowledge of onchocerciasis (i.e. only 35.4 % of the study participants had good knowledge),and Similarly, majority of the study subjects had poor attitude and practice about onchocerciasis (i.e. only 24.1 and 22.8 % study subjects had good attitude and good practice about onchocerciasis, respectively). This finding is also consistent with the findings of the study conducted in Sequa area, Southwest Ethiopia [19]; the study conducted in Quara district, Northwest Ethiopia [21] and the study conducted in Homa District, Western Ethiopia [20]. This is probably due to shortage of health education at the community level and the CDDs may not be properly trained about onchocerciasis due to negligence of health extension workers to supervise the CDDs in delivering health education, and/or excluding the community interventions for onchocerciasis in the health extension package.

In this study, majority of the study subjects had been found to be very devoted and highly volunteer to serve their community with high treatment coverage. In addition to this, majority of the participants knew the way of drug distribution (house to house) which is consistent with the findings of the study conducted in Sequa area, Southwest Ethiopia [19].

Majority of the study subjects in this study indicated that the community participation and perception on the program were high. In addition to this, majority of participants recommended to have incentive to them during the period of drug distribution similar to other health programs. The findings are consistent with the findings of the study conducted in Sequa area, Southwest Ethiopia [19] and the study conducted in Homa District, Western Ethiopia [20].

The limitations of this study were: the study was not supported by qualitative methods like focus group discussions and epidemiological studies. In spite of this limitation, this study provides an important information regarding knowledge, attitude and practice of CDDs towards onchocerciasis and CDTI chemotherapy for onchocerciasis control, identifying perceptions of the CDDs which hinder to distribute the drug in the district, and base line information for the Quara District Health Office for planning, monitoring and evaluation of the on-going onchocerciasis control.

## Conclusion

In conclusion, though all of the CDDs in the study are familiar with onchocerciasis, most of them lack information on the correct causative agent, mode of transmission and prevention of onchocerciasis with conspicuous misconceptions in all issues. This could affect the success of the CDTIP in the present study area. Hence, this study revealed the need for increasing the rate of awareness about onchocerciasis in the area through community-based campaigns during drug distribution with especial focus on females and age group less than 35 years is very important for the effectiveness of the program. This will be important to improve acceptance and support of the CDTI. Health providers should be properly provide refreshment training especially for CDD individuals who serve less than 5 years and evaluated the KAP of the CDDs generally on onchocerciasis and CDTIP. Provide incentives to CDDs to promote the success of drug distribution. Development of health education materials should focus on causative agent, mode of transmission, and prevention of onchocerciasis information in order to ensure better understanding of individuals about the disease.

## Additional file

10.1186/s13104-016-2010-x Questionnaire.
