# Supplementary material for: Knowledge, attitude and practice of community drug distributors’ about onchocerciasis and community directed treatment with ivermectin in Quara district, North Western Ethiopia
Source: BMC Res Notes. 2016 Apr 6;9:206. doi: 10.1186/s13104-016-2010-x (PMC4822301; doi:10.1186/s13104-016-2010-x)
Supplement: Supplementary file 1 — 10.1186/s13104-016-2010-x Questionnaire. [file 13104_2016_2010_MOESM1_ESM.doc]

# Additional file

**Questionnaire**

Addis Ababa University

Aklilu lemma institute of pathobiology

assessment of knowledge, attitude, and practice of onchocerciasis and community directed treatment ivermectin chemotherapy of onchocerciasis control program among CDDs of Quara District.

01. Name of the kebeles ______________

02. Questionnaire identification number_________________

**INTRODUCTION:**

My Name is _______________________. I came from __________________. I am student of graduate studies of Addis Ababa University. I would like to inform you that I will interview you and you will have a short discussion concerning this study. Before we go to our discussion, I will request you to listen carefully to what I am going to read to you about the purpose and general condition of the study and tell me whether you agree or disagree to participate in this study.

**Consent form**

The purpose of this study is to assess knowledge, attitude and practice of community to wards Onchocerciasis and CDTI chemotherapy for Onchocerciasis control. you are selected to be one of the participants in the study. The study will be conducted through interviews. I will ask you for a little of your time, about 15-30 minutes, to help us in this study. In the end, it is hoped that the information you give us could help to solve problems related to knowledge, attitude and practice of community to wards Onchocerciasis and CDTI chemotherapy for Onchocerciasis control. The interview involves personal factors, socio economic factors and provider related factors. I would like to assure you that this privacy should strictly be maintained throughout. A code number will identify every participant and no name will be used. You don’t have to discuss issues that you do not want to and you may end the interview any time. If you want to withdraw from the study any time along the study process you will not be obliged to continue or give reasons for doing so. Refusing to participate or withdrawing from the study along the process will not have any consequences on you. Your responses to any of the questions will not be given to anyone else and no reports of the study will ever identify you. If a report of results is published, only information about the total group will appear.

The interview is voluntary. Your participation/ non-participation, or refusal to respond to the questions will have no effect now or in the future on services that you or any member of your family may receive from any service providers are you willing to participate in this study?

1. Yes 2. No

Thanks in advance for whatever cooperation you do

Questionnaires to assess knowledge, attitude and practice (KAP) of CDDs towards onchocerciasis and CDTI chemotherapy for Onchocerciasis control

| NO | | | Questions | | --- | | | Coding categories |  | | --- | --- | | | Code of the response | |
| --- | --- | --- | --- | --- | --- | --- | --- | --- | --- |
| Socio demographic related questions | | | | | | |
| 01 | Gender of respondent | | | - 1. Male   2. Female | | /…./ |
| | 02 | | --- | | Age (year) | | | 1. 18-24 2. 25-49 3. 50-64 4. 65+ | | /…../ |
| 03 | Ethnic group | | | 1. Amhara 2. Agewu 3. Gumuz 4. others-------------- | | /……./ |
| 04 | Marital status | | | 1. Unmarried 2. Married 3. Divorced 4. Widowed | | /……./ |
| 05 | Religion | | | 1. Orthodox 2. Catholic 3. Protestant 4. Muslim 5. Others-------------- | | /…./ |
| 06 | **Education level** | | | | 1. Illiterate 2. can read 3. primary (1-8) 4. secondary(9-12) 5. Diploma and above | | --- | | | /……./ |
| 07 | what is your occupation | | | 1. Governmental employed 2. Private employed 3. Unemployed 4. Student 5. farmer 6. Merchant 7. others (specify it------) | | /…./ |
| 08 | Family size | | | 1. 1-4 2. 5-8 3. 9 or more | | \..........\ |
| 09 | Have you ever heard about the diseases called onchocerciasis? | | | 1. yes 2. No | | \..........\ |
| 10 | If your answer for Q_9_ is yes, What is the cause of the Onchocerciasis? | | | 1. Filarial worm 2. Black(river) fly 3. Mosquito 4. Living in poor environmental sanitation 5. Poor personal hygiene 6. Sun Scorching 7. Witchcraft 8. Hereditary 9. Eating contaminated food 10. Being not vaccinated 11. Unknown cause 12. Other (specify it…….) | | \..........\ |
| 11 | Do you think oncho transmits? | | | 1. Yes 2. No 3. I do not known | | \..........\ |
| 12 | If your answer for Q_11_ is yes , what is the mode of transmission of the disease | | | | 1. Black fly bite |  | | --- | --- | |  |  | | 1. contact with infected person |  | | 1. Mosquito bite |  | | 1. Through breath |  | |  |  | | 1. Sharing clothes |  | | 1. Sexual contact 2. I do not know 3. Other (specify it ……) | | | | \..........\ |
| 13 | Have you/your families ever been sick from onchocerciasis? | | | 1. yes 2. No 3. I do not remember | | \......\ |
| 14 | What are the signs and symptoms of the disease? | | | 1. Itching 2. Edema 3. Skin change 4. I do not know 5. Other (specify it ………) | | \......\ |
| 15 | Is Onchocerciasis a serious disease? | | | 1. yes 2. No 3. I do not know | | \.........\ |
| 16 | Do you think onchocerciasis needed treatment? | | | 1. yes 2. No 3. I do not know | | \......\ |
| 17 | If your answer for Q_16_ is yes, what type of treatment is used? | | | 1. modern 2. Traditional (specify it ..) 3. I don’t know | | \......\ |
| 18 | If modern, which drug is needed to treat the disease? | | | 1. ivermectin/mectizan 2. Albendazole 3. Others (specify….) | | /..........\ |
| 19 | Do you think oncho is preventable disease? | | | 1. yes 2. No 3. I do not know | | \......\ |
| 20 | If your answer for Q_19_ is yes, What do you do to prevent Onchocerciasis? | | | - 1. Avoiding river bathing   2. Wearing protective clothes   3. Taking drug   4. Using bed net   5. Environmental sanitation   6. Personal hygiene   7. I do not know   8. Others (specify….) | | \..........\ |
| 21 | If your answer for Q_20_ is Wearing protective clothes, in what way is used? | | | - 1. In the lower extremities ( below the knees)   2. around head & shoulders   3. Others (specify it…) | | \......\ |

**Questionnaires to assess knowledge, attitude and practice (KAP) of CDDs towards CDTI** chemotherapy for Onchocerciasis control

|  | | | |
| --- | --- | --- | --- |
| 22 | For how long do you served as CDD | 1. 1year 2. 2 years 3. 3years 4. 4years 5. 5 years and above |  |
| 23 | How is the mechanism of distribution of the drug? | 1. At center place 2. House to house | \..........\ |
| 24 | Are you voluntary to serve the community? | 1. Yes 2. No | \..........\ |
| 25 | If your answer for Q_24_ is no, what is your reason for not serving? | - 1. Lack of support from government   2. Lack of commitment   3. Others (specify it…..) | \..........\ |

| 26 | How is the coverage of drug distribution in the village? | - 1. 100%   2. Partially | \..........\ |
| --- | --- | --- | --- |
| 27 | Did the drug have serious side effects on the community? | - 1. Yes always   2. Yes few   3. No | \..........\ |
| 28 | If your answer for Q_27_ is yes , how do you detect ? | 1. From Individual report 2. Making round visit 3. Others (specify it….) | \..........\ |
|  |  |  |  |
| 29 | Is there anyone who interrupts the treatment? | 1. Yes 2. No 3. I do not know | \..........\ |
| 30 | If your answer for Q_29_ is yes, what is the reason for interrupt the treatment? | 1. Fear of the side effect of the drug 2. Others (specify it….) | \..........\ |
| 31 | What is the status of community participation? | 1. High 2. Low 3. I don’t know | \..........\ |
| 32 | What is the perception of the community for CDTI? | 1. Very good 2. Good 3. poor | \..........\ |
| 33 | What is your recommendation to continue the program? | 1. Incentive is needed 2. Support from health profession 3. Others (specify it……) | \..........\ |
